# Origin, Divergence, and Phylogeny of Asexual *Epichloë* Endophyte in *Elymus* Species from Western China

**DOI:** 10.1371/journal.pone.0127096

**Published:** 2015-05-13

**Authors:** Hui Song, Zhibiao Nan

**Affiliations:** 1 Key Laboratory of Grassland Agro-Ecosystems, Lanzhou, 730020, P. R. China; 2 College of Pastoral Agriculture Science and Technology, Lanzhou University, Lanzhou, 730020, P. R. China; Saint Mary's University, CANADA

## Abstract

Asexual *Epichloë* species are likely derived directly from sexual *Epichloë* species that then lost their capacity for sexual reproduction or lost sexual reproduction because of interspecific hybridization between distinct lineages of sexual *Epichloë* and/or asexual *Epichloë* species. In this study we isolated asexual *Epichloë* endophytes from *Elymus* species in western China and sequenced intron-rich regions in the genes encoding β-tubulin (*tubB*) and translation elongation factor 1-α (*tefA*). Our results showed that there are no gene copies of *tubB* and *tefA* in any of the isolates. Phylogenetic analysis showed that sequences in this study formed a single clade with asexual *Epichloë bromicola* from *Hordeum brevisubulatum*, which implies asexual *Epichloë* endophytes that are symbionts in a western Chinese *Elymus* species likely share a common ancestor with asexual *E*. *bromicola* from European *H*. *brevisubulatum*. In addition, our results revealed that asexual *E*. *bromicola* isolates that are symbionts in a western Chinese *Elymus* species and sexual *Epichloë* species that are symbionts in a North American *Elymus* species have a different origin. Further analysis found that *Epichloë* species likely originated in Eurasia. In addition, the results support the hypothesis that migratory birds or humans might have aided the dispersal of these fungal endophytes to other continents.

## Introduction

Fungi species in the genus *Epichloë* (Clavicipitaceae, Ascomycota) and closely related asexual *Epichloë* species are common endophytes of cool-season grasses in the subfamily Pooideae [1,2]. *Epichloë* species often provide numerous benefits to their hosts, such as increased tolerance to drought [3–5], disease-resistance [6], resistance to herbivory and parasitism [7,8], and enhanced above-ground and below-ground vegetative and reproductive growth [9]. Previous studies have confirmed that certain alkaloids in *Epichloë* species play a crucial role in pasture persistence. For example, lolines and peramine are toxic and confer significant deterrent activity against insect pests [10,11].

Many new *Epichloë* species have recently been identified based on interfertility tests, morphology, molecular phylogenetics and host specificity [12]. However, Leuchtmann et al. [12] re-examined the classification of several described sexual *Epichloë* and asexual *Epichloë* species and varieties, and proposed new combinations and states. This resulted in the acceptance of 43 new *Epichloë* taxa, including species, subspecies and varieties [12].

Asexual *Epichloë* species elicit no visible symptoms of infection and are efficiently transmitted through host seeds (vertical transmission) [13]. In contrast, sexual *Epichloë* species transmit to new hosts through the stigmata based on horizontal transmission [14]; however, sexual *Epichloë* species can also be vertically transmitted through seeds [15–17]. Although some asexual *Epichloë* species are considered incapable of horizontal transmission [18], it has been confirmed that the asexual *Epichloë poae* is capable of horizontal transmission through conidia [19]. In addition, the success of vertical and horizontal transmission can depend on environmental conditions [20,21]. For example, humidity may be critical for successful infection by contagious spores [22], whereas successful establishment of vertically infected seedlings may depend on soil moisture [23]. Molecular phylogenetic analyses of endophytes suggest that host jumps are common events between different species and genera of Pooideae [24,25], which is consistent with host generalism of other members of Hypocreales that are recognized for inter-kingdom host jumps with a high frequency [26].

Researchers have determined that there are two possible origins of asexual *Epichloë* species. The first hypothesis suggests that asexual *Epichloë* species evolved from sexual *Epichloë* species and then lost the ability to sexually reproduce as determined from phylogenetic analyses of β-tubulin (*tubB*) and rDNA-ITS sequences [27]. Alternatively, asexual *Epichloë* species may have derived from interspecific hybrids between sexual *Epichloë* and/or asexual *Epichloë* species [28,29]. There is evidence that many of the recognized asexual *Epichloë* species are hybrids [12]. Hybrids might be selected because hybridization would relieve the effects of Muller’s ratchet, the irreversible accumulation of deleterious mutations that cannot be purged by recombination in clonal species [30]. Hybridization would also allow for the accumulation of genes for alkaloid production, a defence that improves the host's fitness and, owing to vertical transmission, the fitness of the fungus itself [30].

*Elymus* L. is the largest genus of grasses in the tribe Triticeae (Poaceae), which contains about 150 perennial species distributed across temperate zones throughout most of the world [31], except for Africa and Antarctica [32]. In the present study, we isolated 16 asexual *Epichloë* endophytes from western Chinese *Elymus* species, and cloned their encoding β-tubulin (*tubB*) and translation elongation factor 1-α (*tefA*) housekeeping gene sequences. The goals of this study were to (1) elucidate the origin and divergence of 16 asexual *Epichloë* endophytes from western Chinese *Elymus* species; (2) compare sexual *Epichloë* species from North American *Elymus* species and the 16 asexual *Epichloë* endophytes from western China; and (3) estimate the geographical origin and gene-flow of *Epichloë* species.

## Materials and Methods

### Ethics statement

No specific permissions were required since in this study we only collected a limited amount of seeds and stalks from native grassland, and this grassland is not privately-owned or protected in any way. Our field study did not involve any endangered or protected species.

### Plant collection and endophyte isolation

In the present study, between 2011 and 2013 we collected 871 individual plants of nine polyploid *Elymus* species from western China, including the provinces of Ningxia, Gansu, Qinghai, Sichuan, Xinjiang and Tibet. We examined endophyte-infected grasses using the aniline blue coloring (0.1% aqueous) method [33] and isolated fungal endophytes on potato dextrose agar, incubated in darkness at 25°C for four weeks [34].

### DNA extraction, amplification and sequencing

Endophyte total genomic DNA was extracted from fresh mycelia using the HP fungal DNA kit (OMEGA, Beijing, China). Intron-rich portions of the housekeeping genes β-tubulin (*tubB*) and translation elongation factor 1-α (*tefA*) were amplified by polymerase chain reaction (PCR) using universal primers according to the previous study of Moon et al. [15]. The primers in this study were as follows: tub2-exon 1d-1: GAGAAAATGCGTGAGATTGT and tub2-exon 4u-2: GTTTCGTCCGAGTTCTCGAC; and tef1-exon 1d-1: GGGTAAGGACGAAAAGACTCA and tef1-exon 5u-1: CGGCAGCGATAATCAGGATAG. The PCR standard reaction was carried out with 0.5 μ l of genomic DNA, 1 μ l of each primer (10 pmol), 12.5 μ l 2 × taq master-mix and RNAse-free water added to a total of 25 μ l. The *tubB* PCR cycling program was as follows: 94°C for 5 min, followed by 35 cycles of 94°C for 30 s, 45°C for 45 s and 72°C for 2 min, followed by a final extension at 72°C for 10 min. The *tefA* PCR cycling program was as follows: 94°C for 5 min, followed by 35 cycles of 94°C for 30 s, 55°C for 45 s and 72°C for 2 min, followed by a final extension at 72°C for 10 min. PCR products were cloned into the pMD 18-T vector (TaKaRa, Dalian, China) based on the manual. Five positive clones per genes were sequenced by TaKaRa Biotechnology Co. Ltd (Dalian, China).

Sequences were deposited in GenBank: *tefA*: KJ585716- KJ585730; *tubB*: KJ585731- KJ585745. In addition, 43 unique taxa in the *Epichloë* sequences of endophyte *tubB* and *tefA* genes were obtained from GenBank (S1 Table).

### Data analysis

Endophyte *tubB* and *tefA* sequences were aligned using the MAFFT 7.0 program [35] and alignments were adjusted by eye. Maximum parsimony (MP) trees were constructed in the PAUP 4.0b10 package [36]. MP trees were estimated using a heuristic search with tree bisection-reconnection (TBR) branch swapping and 100 random addition replicates. Alignment gaps were treated as missing information. Nucleotide substitutions were unordered and unweighted. Maximum likelihood (ML) trees were constructed in the MEGA 6.0 program [37]. The optimal model of nucleotide evolution was HKY+G for *tubB* and *tefA*, according to MrModeltest 2.3 [38] and this model was used in the ML analysis. ML heuristic searches were performed with 100 random addition sequence replications and TBR branch swapping algorithm. Bootstrap support values were calculated with 1000 replicates.

Nucleotide diversity was calculated using Tajima's π [39] and Watterson's θ [40] statistics. The Tajima's π measure quantifies the mean percentage of nucleotide differences among all pairwise comparisons for a set of sequences; whereas, Watterson's θ is simply an index of the number of segregating (polymorphic) sites. To tests the neutral evolution, Tajima's D and Fu and Li's D statistics were inferred as described by Tajima [39] and Fu and Li [41]. The software program DnaSP 5.0 [42] was used to perform the above calculations.

Median-joining (MJ) network analysis was applied to display the genealogical relationships between taxa [43,44]. Previously there has been very little information published that related to the phylogenetic network of the endophytes. In the present study, we determined the haplotype of sequences excluding the outgroup based on the DnaSP 5.0 program [42] and constructed a network of the endophytes using the Network 4.1 program (Fluxus Technology Ltd, Clare, Suffolk, UK).

## Results

### Sequence variation

Sixteen asexual *Epichloë* endophytes were isolated from western Chinese *Elymus* species (S1 Table). The fragment sequence of the *Epichloë tubB* sequence in this study includes four exons and three introns, while the fragment sequence of the *Epichloë tefA* sequences contains three exons and three introns. Furthermore, no gene copies of *tubB* and *tefA* were observed in any of the isolates. In addition, *tubB* and *tefA* sequence of isolates NI_201207 and NI_201209 could not be obtained (S1 Table). Analyses of the *Epichloë tubB* and *tefA* sequences found that the exon sequences are more conserved than the intron sequences (data not shown). More importantly, previous studies [15,45] used intron sequences to provide insight into the phylogenetic relationships of endophytes. Therefore we also used the intron sequences.

The length of the *tubB* intron sequences in this study varied from 244 to 403 bp. The length of the alignment of the *tubB* intron sequences was 495 bp, including 62 invariable sites and 115 variable sites, 55 of which were parsimony informative sites. The length of the *tefA* intron sequences varied from 348 to 551 bp. The length of the alignment of the *tefA* intron sequences was 732 bp, including 90 invariable sites and 136 variable sites, 77 of which were parsimony informative sites.

We estimated the haplotypes and nucleotide polymorphisms of the *Epichloë* species in North American and western Chinese *Elymus* species. In the *tubB* sequences, the number of haplotypes (6) in the asexual *Epichloë* endophytes from western Chinese *Elymus* species was higher than the number of haplotypes (4) in the *Epichloë* species from North American *Elymus* species. The number of polymorphic sites (10) in the asexual *Epichloë* endophytes from western Chinese *Elymus* species is much lower than the number of polymorphic sites (29) in the *Epichloë* species from North American *Elymus* species (Table 1). The nucleotide diversity Tajima's π and Watterson's θ values in the asexual *Epichloë* endophytes from western Chinese *Elymus* species were 0.0038 and 0.00881, respectively, while in the *Epichloë* species from North American *Elymus* species they were 0.0326 and 0.0332, respectively (Table 1), indicating that the nucleotide diversity in *Epichloë* species from North American *Elymus* species is higher than the nucleotide diversity in asexual *Epichloë* endophytes from western Chinese *Elymus* species. The Tajima's D and Fu and Li's D values of asexual *Epichloë* endophytes from western Chinese *Elymus* species were -2.0146 (*p* < 0.05) and -2.4555 (*p* < 0.05), respectively, while for the *Epichloë* species from North American *Elymus* species they were -0.2916 (*p* > 0.1) and -0.1983 (*p* > 0.1), respectively, indicating that the asexual *Epichloë* endophytes from western Chinese *Elymus* species had a significant departure from neutrality.

**Table 1:** Estimates of nucleotide diversity and selection statistics for *tubB* and *tefA* sequences in *Epichloë* endophytes from western Chinese and North American *Elymus* species.

	N	h	n	s	π	θ	Tajima's D	Fu and Li's D
Western Chinese *Elymus* species, *tubB*	15	6	381	10	0.0038	0.0081	-2.0146 (*p*<0.05)	-2.4555 (*p*<0.05)
North American *Elymus* species, *tubB*	7	4	393	29	0.0326	0.0332	-0.2916 (*p*>0.1)	-0.1983 (*p*>0.1)
Western Chinese *Elymus* species, *tefA*	15	10	496	12	0.0052	0.0075	-1.1814 (*p*>0.1)	-1.8640 (*p*>0.1)
North American *Elymus* species, *tefA*	7	5	546	48	0.0450	0.0402	0.4240 (*p*>0.1)	0.7607 (*p*>0.1)
Western Chinese *Elymus* species, *tubB*	15	6	381	10	0.0038	0.0081	-2.0146 (*p*<0.05)	-2.4555 (*p*<0.05)
North American *Elymus* species, *tubB*	7	4	393	29	0.0326	0.0332	-0.2916 (*p*>0.1)	-0.1983 (*p*>0.1)
Western Chinese *Elymus* species, *tefA*	15	10	496	12	0.0052	0.0075	-1.1814 (*p*>0.1)	-1.8640 (*p*>0.1)
North American *Elymus* species, *tefA*	7	5	546	48	0.0450	0.0402	0.4240 (*p*>0.1)	0.7607 (*p*>0.1)

Note: N: the number of sequences analyzed; h: the number of haplotypes; n: the number of the sites (excluding sites with gaps and missing data); s: number of polymorphic sites; π (Tajima’s π): nucleotide diversity; θ (Watterson’s θ): the diversity based on the number of polymorphic sites.

In the *tefA* sequences, the number of haplotypes (10) in the asexual *Epichloë* endophytes from western Chinese *Elymus* species was much higher than the number of haplotypes (5) in the *Epichloë* species from North American *Elymus* species (Table 1). The number of polymorphic sites (12) in the asexual *Epichloë* endophytes from western Chinese *Elymus* species was much lower than the polymorphic sites (48) in the *Epichloë* species from North American *Elymus* species. The nucleotide diversity or Tajima's π value of the asexual *Epichloë* endophytes (0.0052) from western Chinese *Elymus* species is lower than the Tajima's π value in the *Epichloë* species (0.0450) from North American *Elymus* species. The nucleotide diversity or Watterson's θ value of the asexual *Epichloë* endophytes (0.0075) from western Chinese *Elymus* species is lower than the Watterson's θ value in the *Epichloë* species (0.0402) from North American *Elymus* species (Table 1). The Tajima's D value was -1.1814 (*p* > 0.1) for the asexual *Epichloë* endophytes from western Chinese *Elymus* species and it was 0.4240 (*p* > 0.1) for the *Epichloë* species from North American *Elymus* species. The Fu and Li's D value was -1.8640 (*p* > 0.1) for the asexual *Epichloë* endophytes from western Chinese *Elymus* species, and it was 0.7607 (*p* > 0.1) for the *Epichloë* species from North American *Elymus* species, indicating that different selection pressures affected the asexual *Epichloë* endophytes from western Chinese *Elymus* species and *Epichloë* species from North American *Elymus* species.

### Phylogenetic analyses of *tubB* and *tefA* sequences

To reveal the phylogenetic relationships between asexual *Epichloë* endophytes from western Chinese *Elymus* species and other *Epichloë* species, we constructed phylogenetic trees with maximum parsimony (MP) and maximum likelihood (ML) methods using *tubB* and *tefA* sequences. Parsimony analysis of the *tubB* sequences yielded a tree length of 470 (CI = 0.685 and RI = 0.938; S1 Fig) and parsimony analysis of the *tefA* sequences yielded a tree length of 507 (CI = 0.751 and RI = 0.946; S2 Fig). The MP method produced a tree topology nearly identical to the ML trees. We only showed MP tree in S1 and S2 Figs, respectively.

**Fig 1:**
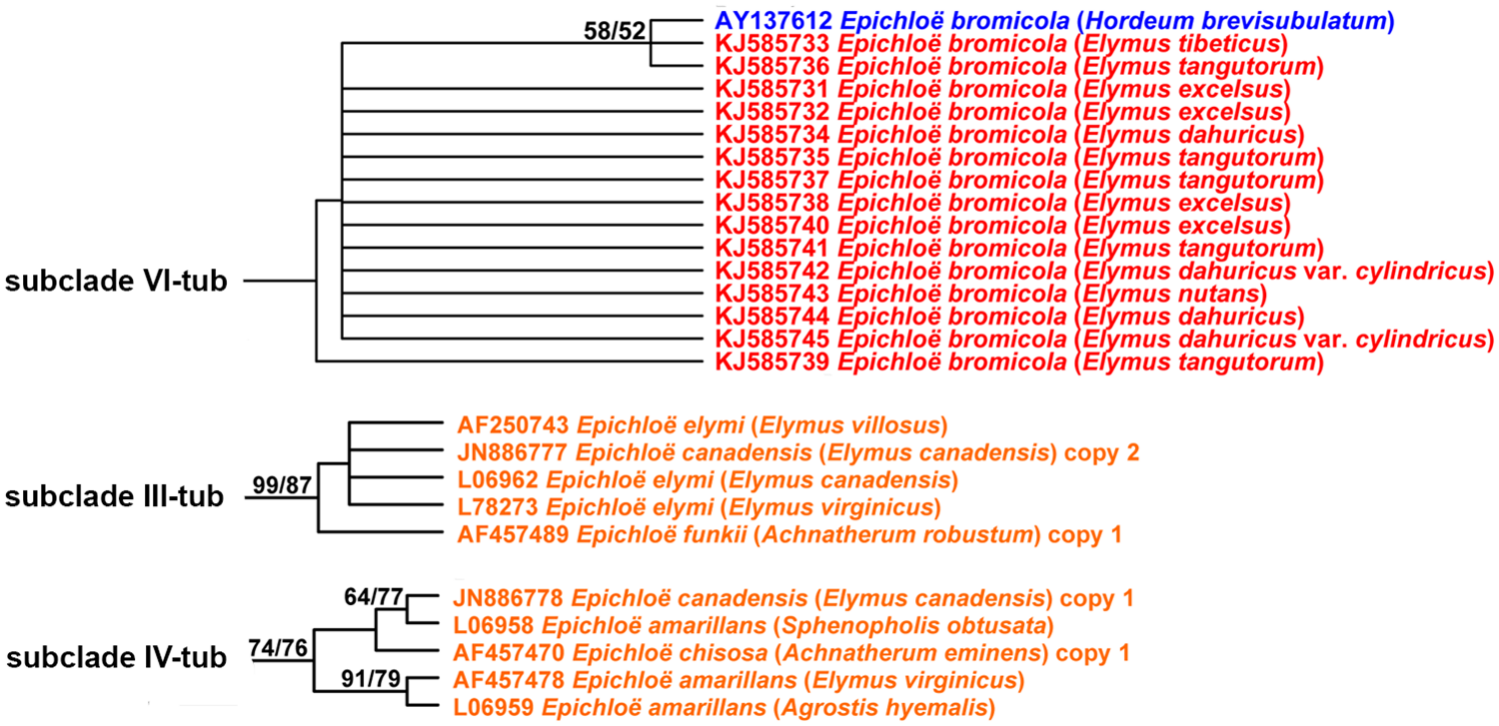
Maximum parsimony (MP) phylogenetic relationships of *Epichloë* species based on intron portions of *tubB*. MP trees were constructed in the PAUP 4.0b10 package. MP trees were estimated using a heuristic search with tree bisection-reconnection (TBR) branch swapping and 100 random addition replicates. Alignment gaps were treated as missing information. Nucleotide substitutions were unordered and unweighted. Bootstrap support values were calculated with 1000 replicates. Numbers on the branches are bootstrap values. Branches with bootstrap values >50% are shown. Maximum likelihood (ML) bootstrap values are listed first on each branch, followed by MP bootstrap values. Red, blue and orange colors indicate the *Epichloë* species from China, Europe and North America, respectively. The partial figure is showed, for the full image please see S1 Fig.

In the *tubB* tree the asexual *Epichloë* endophytes from western Chinese *Elymus* resolved in subclade VI-tub (Fig 1 and S1 Fig). Interestingly, sexual *Epichloë* species from North American *Elymus* species were not in subclade VI-tub, but instead, resolved in subclade III-tub and subclade IV-tub. Although a previous study [27] showed that asexual *Epichloë* species originated from sexual *Epichloë* species and then lost the ability to sexually reproduce, our phylogenetic results found that isolates of western Chinese (asexual endophytes) and North American (sexual endophytes) *Elymus* species have different origins, indicating that they are different species. Subclade VI-tub contained 16 *tubB* sequences, including 15 asexual *Epichloë* endophytes from 15 western Chinese *Elymus* species and one asexual *Epichloë bromicola* from European *Hordeum brevisubulatum*. This suggests that the isolates from western Chinese *Elymus* species likely share a common ancestor with the asexual *E*. *bromicola* from European *H*. *brevisubulatum*. In addition, subclade III-tub contained three sexual *E*. *elymi* from North American *Elymus canadensis*, *El*. *villosus* and *El*. *virginicus*, one asexual *E*. *canadensis* from North American *El*. *canadensis* and one asexual *E*. *funkii* from North American *Achnatherum robustum*. Subclade IV-tub contained three sexual *E*. *amarillans* from North American *Agrostis hyemalis*, *El*. *virginicus* and *Sphenopholis obtusata* respectively, one asexual *E*. *canadensis* from North American *El*. *canadensis* and one asexual *E*. *chisosa* from North American *Ac*. *eminens*.

**Fig 2:**
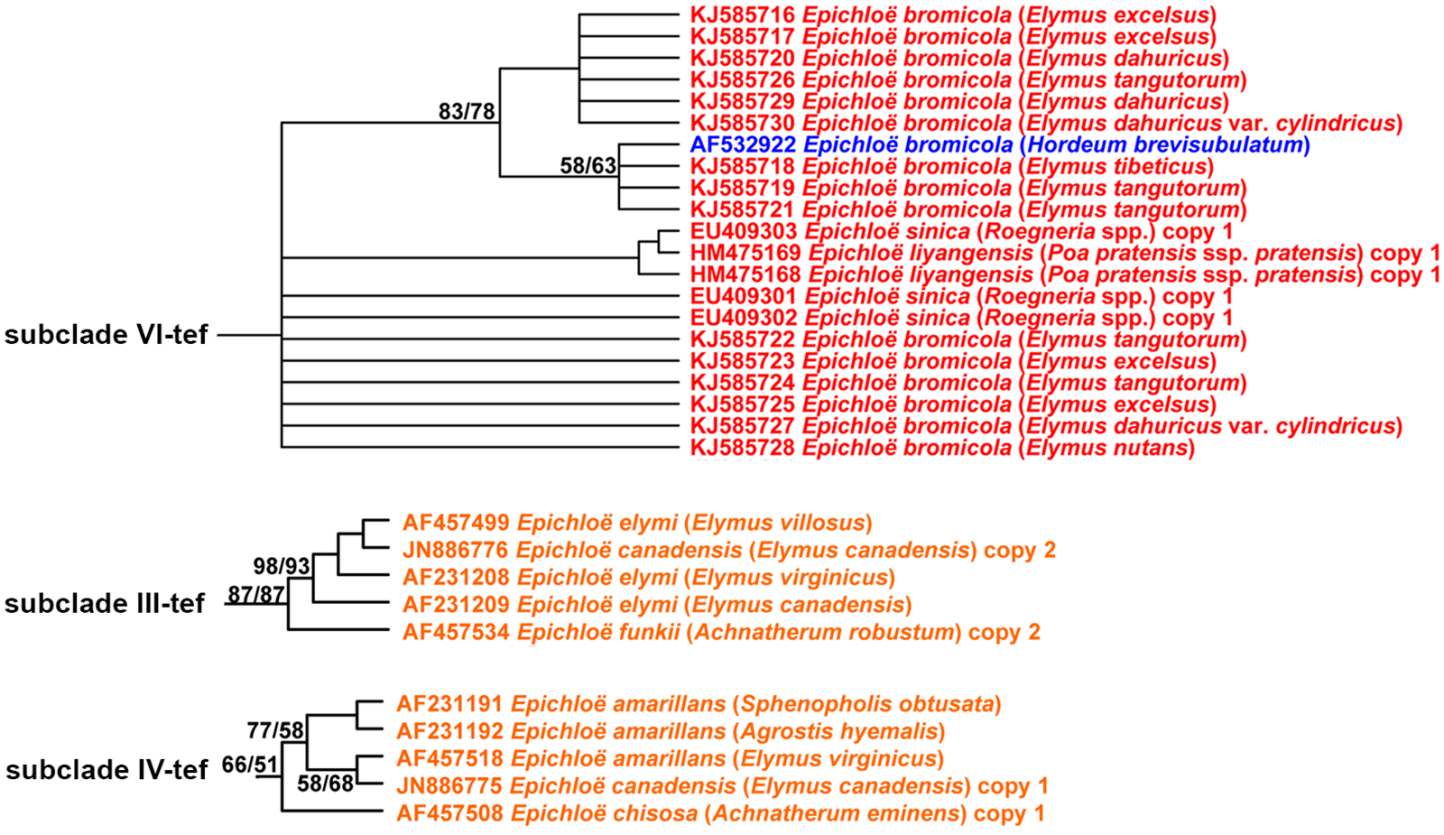
Maximum parsimony (MP) phylogenetic relationships of *Epichloë* species based on intron portions of *tefA*. MP trees were constructed in the PAUP 4.0b10 package. MP trees were estimated using a heuristic search with tree bisection-reconnection (TBR) branch swapping and 100 random addition replicates. Alignment gaps were treated as missing information. Nucleotide substitutions were unordered and unweighted. Bootstrap support values were calculated with 1000 replications. Numbers on branches are bootstrap values. Branches with bootstrap values >50% are shown. Maximum likelihood (ML) bootstrap values are listed first on each branch, followed by MP bootstrap values. Red, blue and orange colors indicate the *Epichloë* species from China, Europe and North America, respectively. The partial figure is showed, for the full image please see S2 Fig.

The topology of the *tefA* tree is consistent with that of the *tubB* tree. Isolates from western Chinese (asexual endophytes) and North American (sexual endophytes) *Elymus* species were grouped in different subclades: subclade III-tef, subclade IV-tef and subclade VI-tef, respectively (Fig 2 and S2 Fig). Subclade VI-tef contained 21 *tefA* sequences, including 15 asexual *Epichloë* endophytes from western Chinese *Elymus* species, three asexual *E*. *sinica* from Chinese *Roegneria* spp., two sexual *E*. *liyangensis* from Chinese *Poa pratensis* ssp. *pratensis* and one asexual *E*. *bromicola* from European *H*. *brevisubulatum*. *E*. *sinica* and *E*. *liyangensis* appear to be hybrids. The results of the *tefA* tree confirmed that the asexual *Epichloë* endophytes from western Chinese *Elymus* species are likely derived from the same ancestor with the asexual *E*. *bromicola* from European *H*. *brevisubulatum*.

Subclade III-tef contained three sexual *E*. *elymi* from North American *El*. *canadensis*, *El*. *villosus* and *El*. *virginicus*, one asexual *E*. *canadensis* from North American *El*. *canadensis* and one asexual *E*. *funkii* from North American *Ac*. *robustum*. Subclade IV-tef contained three sexual *E*. *amarillans* from North American *S*. *obtusata*, *Ag*. *hyemalis* and *El*. *virginicus*, one asexual *E*. *canadensis* from North American *El*. *canadensis* and one asexual *E*. *chisosa* from North American *Ac*. *eminens*. These results suggest that asexual *Epichloë* endophytes from western China and sexual *Epichloë* species from North American *Elymus* species have different origins.

### Network analyses of *tubB* and *tefA* sequences

**Fig 3:**
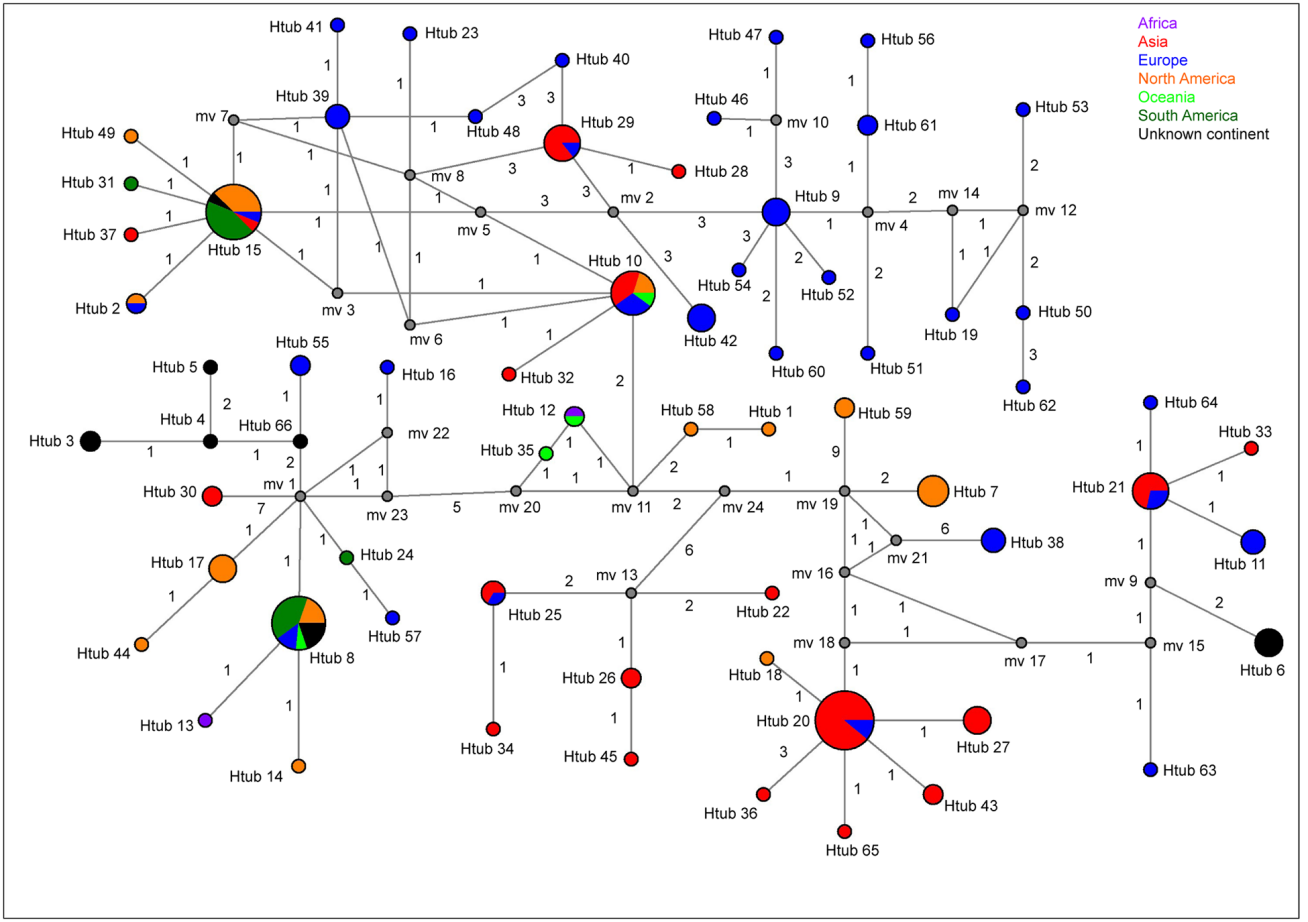
Median-joining (MJ) networks of *tubB* haplotypes from *Epichloë* species. Each circle represents a single haplotype and the circle size is proportional to the number of isolates with that haplotype. Median vectors (mv) indicate missing intermediates of unsampled nodes inferred by the MJ network analysis and the number along the branch shows the number of mutations separating nodes.

Haplotype data can be used to determine ancestral and derived relationships with median-joining (MJ) networks [46], where genetically closely-related taxa are represented as physically closer in the MJ network. The *tubB* MJ network had a haplotype diversity of 0.9629. Fifteen asexual *Epichloë* endophytes from western Chinese *Elymus* species contained two haplotypes: Htub 20 and 65 (Fig 3 and S1 Table). Nine sexual *Epichloë* endophytes from North American *Elymus* species contained three haplotypes: Htub 7, 17 and 44. Htub 20 and 65 were closely related, while Htub 7, 17 and 44 were in different clades (Fig 3). The results also confirm that asexual *Epichloë* endophytes from western Chinese *Elymus* species and sexual *Epichloë* species from North American *Elymus* species have different origins, and the genetic diversity is higher in sexual *Epichloë* species from North American *Elymus* species than in asexual *Epichloë* endophytes from western Chinese *Elymus* species. Htub 65 contained only one *tubB* sequence from asexual *Epichloë* endophytes in western Chinese *Elymus* species, but Htub 20 contained 18 *tubB* sequences from Asia and Europe, including one sexual *Epichloë* species and 17 asexual *Epichloë* endophytes. Among the 17 asexual *Epichloë* endophytes, one asexual *E*. *bromicola* was isolated from the European species, *H*. *brevisubulatum*, and 16 asexual *Epichloë* endophytes were isolated from Asian *Leymus chinensis* (2) and western Chinese *Elymus* species (14). There were 14 asexual *Epichloë* endophytes from western Chinese *Elymus* species, and two asexual *E*. *bromicola* from Asian *L*. *chinensis*. In addition, the asexual *E*. *hordelymi* was isolated from European *Hordelymus europaeus*. Htub 36 contained a sexual *E*. *bromicola tubB* sequence from Asian *El*. *repens*. Htub 36 and 20 were distributed in one clade, indicating closer relationships between these haplotypes.

Htub 7 included three sexual *E*. *elymi* and one asexual *E*. *canadensis*. The three sexual *E*. *elymi* were isolated from North American *El*. *canadensis*, *El*. *villosus* and *El*. *virginicus*. The asexual *E*. *canadensis* was isolated from North American *El*. *canadensis*. Htub 17 and 44 included sequences from sexual *E*. *amarillas* and asexual *E*. *canadensis*. Sexual *E*. *amarillas* was isolated from North American *El*. *virginicus*, and asexual *E*. *canadensis* was isolated from North American *El*. *canadensis*.

**Fig 4:**
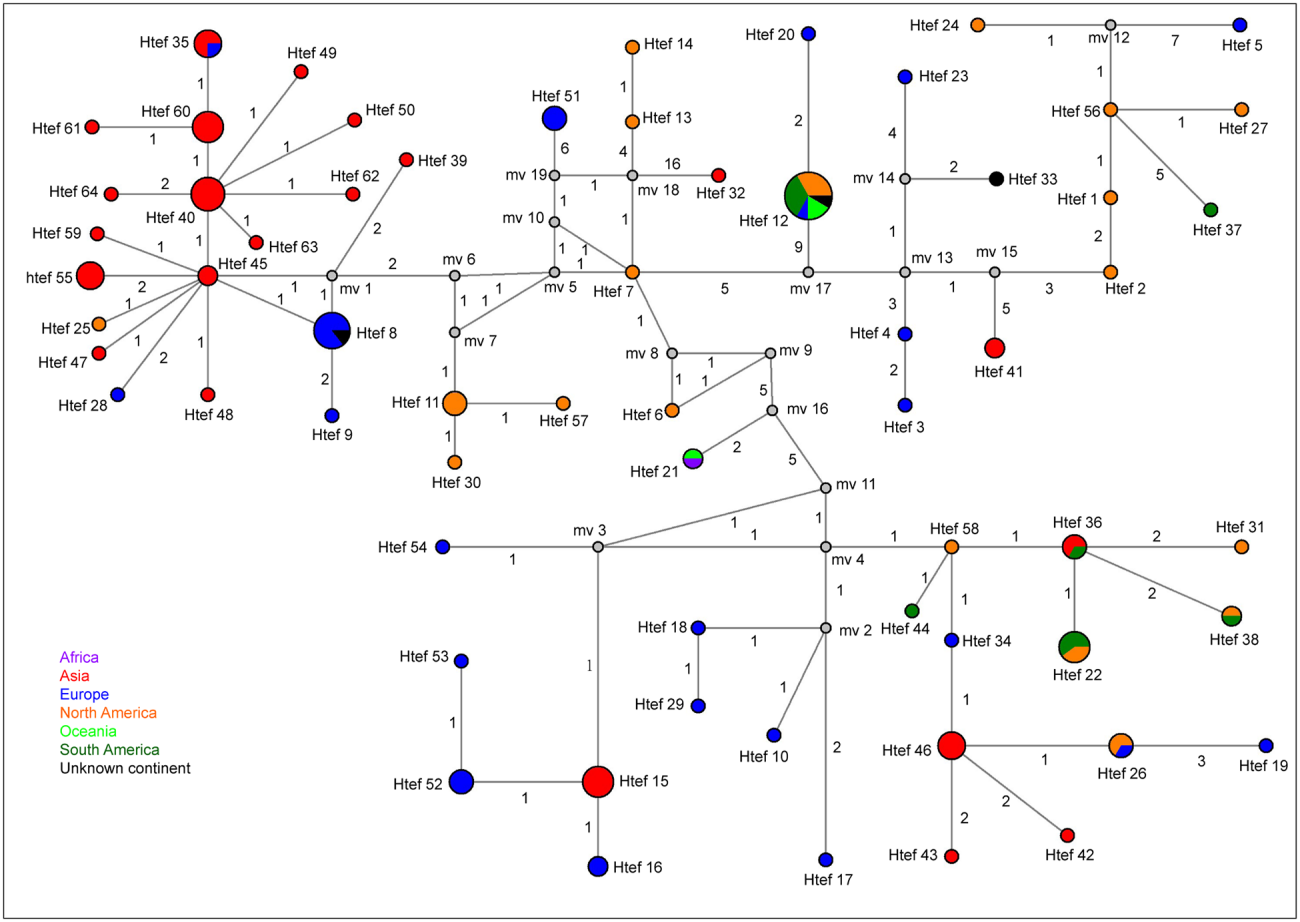
Median-joining (MJ) networks of *tefA* haplotypes from *Epichloë* species. Each circle represents a single haplotype and the circle size is proportional to the number of isolates with that haplotype. Median vectors (mv) indicate missing intermediates of unsampled nodes inferred by the MJ network analysis and the number along the branch shows the number of mutations separating nodes.

The *tefA* MJ network had a haplotype diversity of 0.9770. Fifteen asexual *Epichloë tefA* sequences from western Chinese *Elymus* species contained seven haplotypes, Htef 35, 40, 60, 61, 62, 63 and 64. Htef 61, 62, 63 and 64 only contained one *tefA* sequence each, while Htef 35, 40 and 60 contained three, six and five *tefA* sequences, respectively (Fig 4 and S1 Table). In Htef 35, there were three asexual *Epichloë* endophytes, including two asexual *Epichloë* endophytes isolated from western Chinese *Elymus* species and one asexual *E*. *bromicola* isolated from European *H*. *brevisubulatum*. Htef 40 contained three asexual *Epichloë* endophytes from western Chinese *Elymus* species and three asexual *E*. *sinica* from Asian *Roegneria* spp.. In addition, Htef 60 contained five asexual *Epichloë* endophytes from western Chinese *Elymus* species. The sexual *E*. *bromicola* from *El*. *repens* (Htef 59) is closely related to the asexual *Epichloë* species from western China (Fig 4).

The sexual *Epichloë* species from North American *Elymus* species shared four haplotypes: Htef 11, 27, 56 and 57. Htef 27, 56 and 57 only contained one *tefA* sequence, respectively. Htef 27 was from the North American sexual *E*. *amarillas* (*El*. *virginicus*), and Htef 56 and 57 were from the North American asexual *E*. *canadensis* (*El*. *canadensis*). HapF 11 was from the sexual *E*. *elymi*, whose host plants include *El*. *canadensis*, *El*. *villosus* and *El*. *virginicus* in North America.

## Discussion

Asexual *Epichloë* species are thought to have derived from asexual and/or sexual *Epichloë* species by hybridization [27]. Among the recognized asexual *Epichloë* species, hybrid endophytes (19 of 26 taxa) outnumber non-hybrid endophytes [12]. Moreover, hybrid endophytes are abundant in wild grass populations in temperate areas across the world [47–49]. However, we did not find any gene copies of *tubB* and *tefA* through PCR and sequencing. The *tubB* and *tefA* phylogenetic estimates do not suggest different origins of asexual *Epichloë* endophytes from western Chinese *Elymus* species. Our results reveal that asexual *Epichloë* endophytes from western Chinese *Elymus* species do not hybridize, which indicates that these asexual *Epichloë* species are derived from the same ancestor with *E*. *bromicola* (S1 and S2 Figs).

### Endophyte diversity in North American and western Chinese *Elymus* species

Our *tubB* and *tefA* phylogenetic trees suggest that asexual *Epichloë* endophytes from western Chinese *Elymus* species and sexual *Epichloë* species from North American *Elymus* species have different origins (Figs 1, 2, 3 and 4). Several scenarios would lead to this kind of pattern. Transmission of sexual *Epichloë* species to new hosts occurs through horizontal transmission through the stigmata [14]. In contrast, transmission of asexual *Epichloë* species to the next generation of grass generally occurs in the seed, when hyphae penetrate the developing embryo [15,16]. However, researchers have found that asexual *Epichloë* species could be horizontally spread from plant to plant through conidia produced on the leaf surface [19,24,50]. We found that the asexual *Epichloë* endophytes from western Chinese *Elymus* were closely related to asexual *E*. *bromicola* from European *H*. *brevisubulatum*, asexual *E*. *sinica* from Asian *Roegneria* spp. and sexual *E*. *liyangensis* from Asian *P*. *pratensis* ssp. *pratensis*. This set of relationships indicates that a horizontal transmission mechanism probably exists. In addition, Moon et al. [27] found that transmission of endophytes occurred within the same tribe, but also between different tribes. *Elymus*, *Hordeum* and *Roegneria* are members of the Triticeae tribe, but *P*. *pratensis* ssp. *pratensis* is a member of the Poeae tribe. Although there is only a little information available about horizontal transmission between Triticeae and Poeae, the data presented here and the results described by Moon et al. [27] are sufficient to support horizontal transmission between Triticeae and Poeae.

### Origin and spread of endophytes

The MJ network reflects the ancestral and derived relationships based on haplotype data, but also reveals a clear geographical pattern of distribution to the new or old world [44]. North American (new world) haplotypes of *Epichloë* are nested within old world samples of *Epichloë* species, whereas Asian (old world) haplotypes are grouped within the new world haplotypes (Figs 3 and 4). This pattern indicates that *Epichloë* gene-flow between the new and old world is common. Note that most haplotypes from European and Asian endophytes (old world) are located in MJ network (Figs 3 and 4). These results suggest that European and Asian endophytes have high haplotype diversity. In addition, Europe (19) and Asia (11) had more *Epichloë* speices (70%, 30/43) than any other continent and *E*. *festucae* var. *lolii*, *E*. *typhina*, *E*. *coenophiala* and *E*. *occultans* were introduced from Europe to other places [12]. This finding is consistent with this location being the center of origin for the genus [51] as this is where the greatest species diversity is to be found. In phylogenetic tree, European and Asian endophytes are placed near the root of the clades (S1 and S2 Figs), indicating they are diverged early in the phylogeny. Because of most of Europe and Asia belong to Eurasia from the perspective of geography. So we speculated that *Epichloë* species likely originated in Eurasia.

The *tubB* and *tefA* networks suggest that gene-flow among continents is common. We found that Eurasian endophytes are widely distributed across the globe and that this indicates that Eurasian endophytes “bridge” new and old world endophyte diversity (Figs 3 and 4). The spread of endophytes could be caused by, but is not limited to, the following processes. European endophytes are closely related to American endophytes (Figs 3 and 4) and it is possible that European animals carried endophyte-infected grass plants or seeds while they crossed the Bering land bridge to North America [52]. Another hypothesis is that the spread of endophytes from North America to South America may have been caused by the transport of endophyte-infected grass plants or seeds by rafting or carriage by migratory birds [15]. Endophytes could have spread more easily throughout Eurasia and Africa by human or bird-mediated dispersal. More interestingly, the *Epichloë* endophytes from Oceania are closely related to *Epichloë* from America and Europe (Figs 3 and 4). The most likely distribution scenario to the Oceania is European or American migration to the islands, accompanied by endophyte-infected seeds or plants. This is perhaps not surprising given that asexual *E*. *festucae* var. *lolii* was first introduced to New Zealand in the 1800s, in seeds brought to New Zealand by British immigrants [53]. Furthermore, haplotype Htub 22 and Htef 21 are from African and Oceania endophytes, indicating gene-flow exchange between these two areas. The most likely reason for this pattern is European migration to Oceania, with stop-overs in Africa where there were food and water supplies, and then a selection of endophyte-infected seeds or plants left Africa and were transferred to Oceania.

## Supporting Information

S1 Fig Maximum parsimony (MP) phylogenetic relationships of *Epichloë* species based on intron portions of *tubB*. (PDF)

S2 Fig Maximum parsimony (MP) phylogenetic relationships of *Epichloë* species based on intron portions of *tefA*. (PDF)

S1 Table Taxa used in this study. Note: The ND indicate no sequence detected.
(XLS)
