# Supplementary figures and images for: Origin, Divergence, and Phylogeny of Asexual Epichloë Endophyte in Elymus Species from Western China
Source: PLoS One. 2015 May 13;10(5):e0127096. doi: 10.1371/journal.pone.0127096 (PMC4430518; doi:10.1371/journal.pone.0127096)

Africa  
Asia  
Europe  
North America  
Oceania  
South America  
Unknown continent

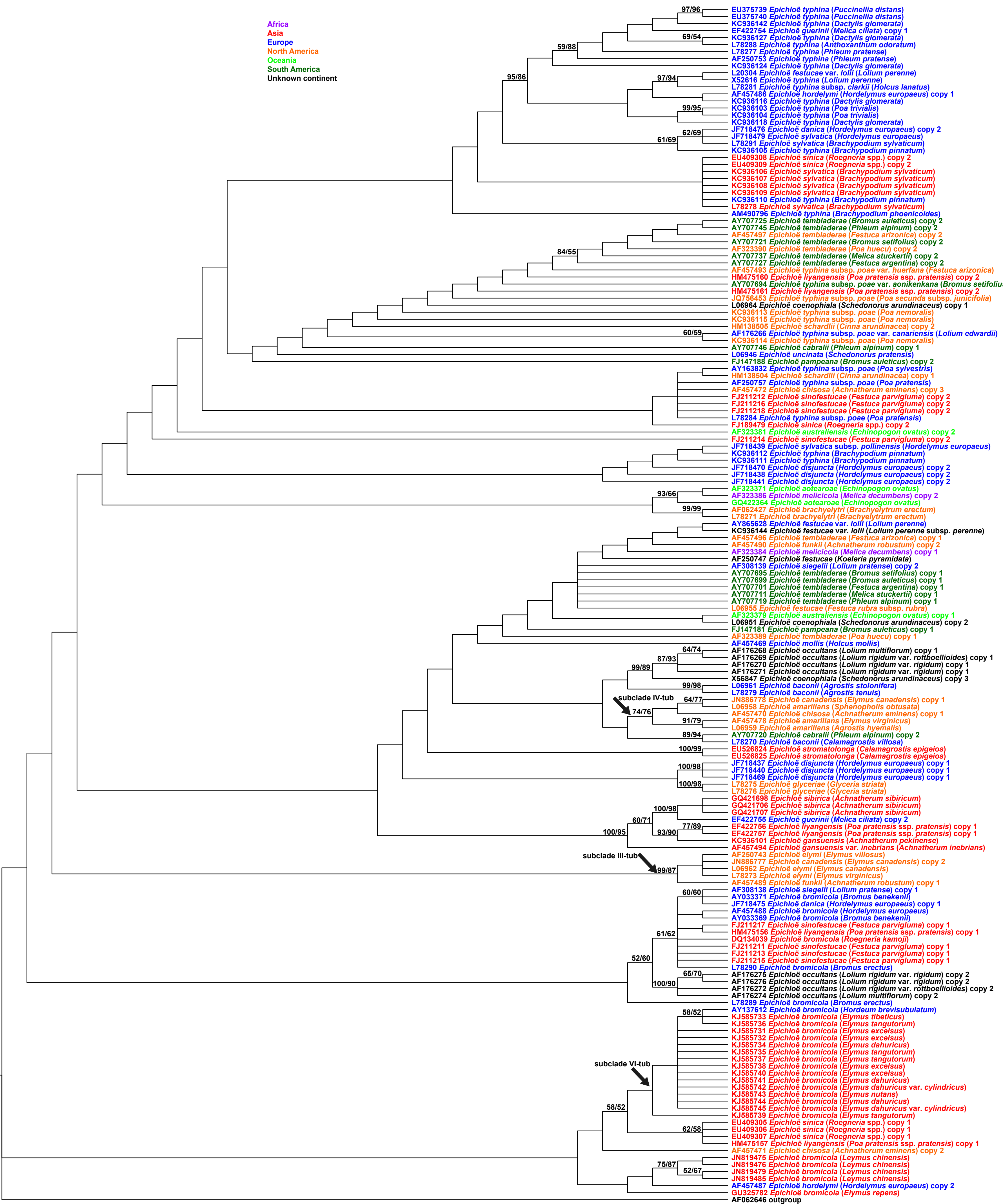

Supplement: S1 Fig — (PDF) [file pone.0127096.s001.pdf]

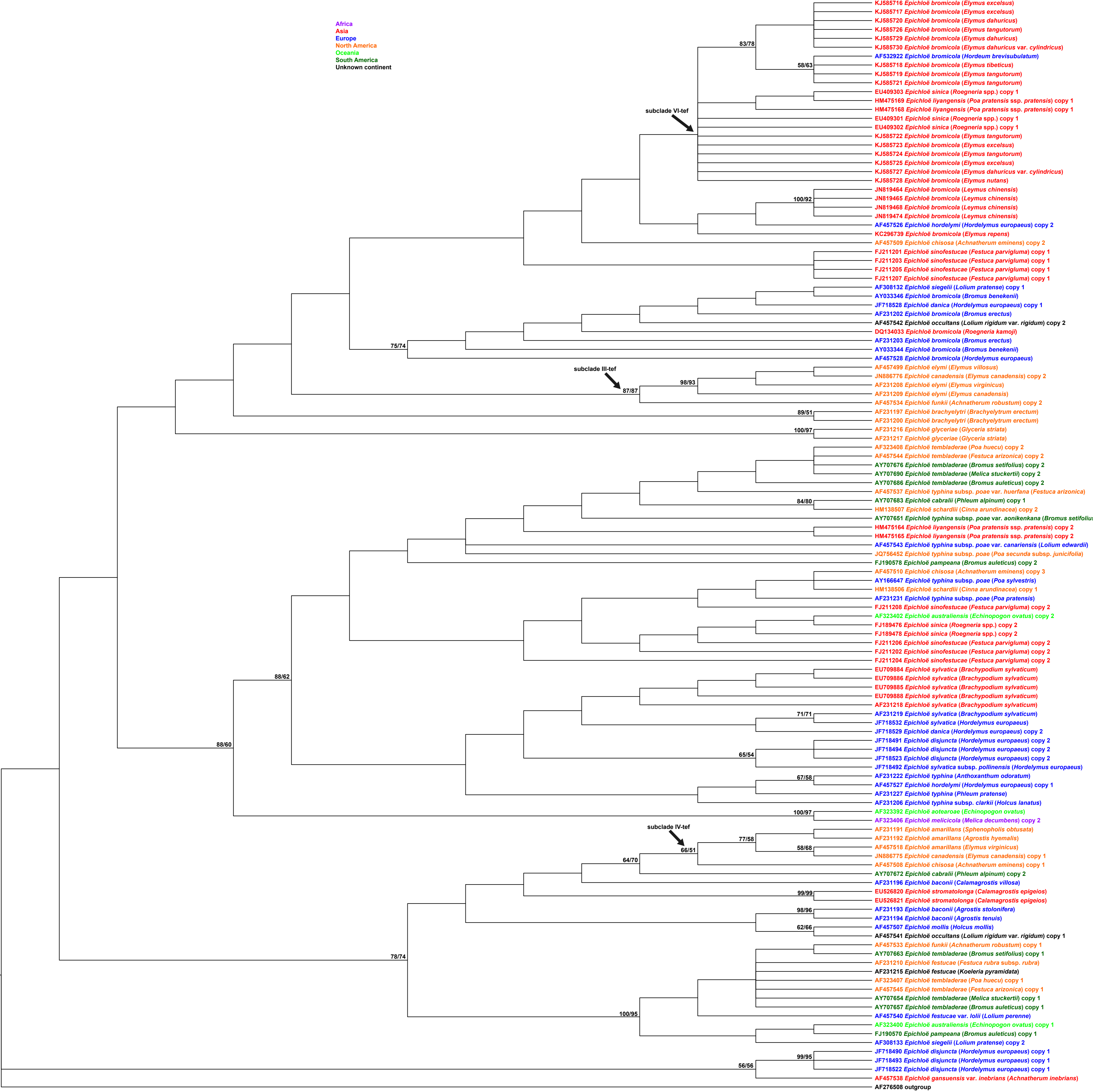

Supplement: S2 Fig — (PDF) [file pone.0127096.s002.pdf]
